# Assessment of Specific Tumoral Markers, Inflammatory Status, and Vitamin D Metabolism before and after the First Chemotherapy Cycle in Patients with Lung Cancer

**DOI:** 10.3390/biology11071033

**Published:** 2022-07-08

**Authors:** Andreea Crintea, Cristina Drugan, Anne-Marie Constantin, Iulia Lupan, Zsolt Fekete, Ciprian Nicolae Silaghi, Alexandra Mărioara Crăciun

**Affiliations:** 1 Department of Medical Biochemistry, Iuliu Hațieganu University of Medicine and Pharmacy, 400349 Cluj-Napoca, Romania; crintea.andreea@umfcluj.ro (A.C.); cdrugan@umfcluj.ro (C.D.); acraciun@umfcluj.ro (A.M.C.); 2 Department of Morphological Sciences, Iuliu Hațieganu University of Medicine and Pharmacy, 400349 Cluj-Napoca, Romania; annemarie.chindris@umfcluj.ro; 3 Department of Molecular Biology and Biotechnology, Babeș-Bolyai University, 400084 Cluj-Napoca, Romania; iulia.lupan@ubbcluj.ro; 4 Department of Oncology, Iuliu Hatieganu University of Medicine and Pharmacy, 400349 Cluj-Napoca, Romania; fekete.zsolt@umfcluj.ro

**Keywords:** neuron-specific enolase, squamous cell carcinoma antigen, neopterin, chitotriosidase, vitamin D, vitamin D receptor, lung cancer

## Abstract

**Simple Summary:**

In patients with lung cancer, serum levels of neuron specific enolase were significantly decreased after the first chemotherapy cycle compared to pre-treatment values. In addition, the post-treatment values of neopterin were lower as well, but with marginal statistical significance. Conversely, circulating levels of chitotriosidase, squamous cell carcinoma antigen, vitamin D, and vitamin D receptor were not significantly modified in response to the first chemotherapy cycle.

**Abstract:**

Background: We aimed to investigate the changes of inflammatory status reflected by serum levels of chitotriosidase (CHT) and neopterin, and how specific tumor markers such as neuron-specific enolase (NSE) and squamous cell carcinoma antigen (SCCA), as well as vitamin D metabolism assessed by vitamin D receptor (VDR) and 25-hydroxy vitamin D_3_ (25OHD_3_), were modified after the first cycle of chemotherapy in patients with lung cancer. Methods: We performed this first pilot study on twenty patients diagnosed with lung cancer by investigating the serum concentrations of CHT, neopterin, NSE, SCCA, VDR and 25OHD_3_ before and after the first cycle of chemotherapy. Results: The post-treatment values of NSE were significantly lower compared to the pre-treatment levels (14.37 vs. 17.10 ng/mL, *p* = 0.031). We noticed a similar trend in neopterin levels, but the difference was only marginally significant (1.44 vs. 1.17 ng/mL, *p* = 0.069). On the contrary, the variations of circulating SCCA, CHT, neopterin, VDR and 25OHD_3_, before and after treatment, did not reach statistical significance. Conclusion: Only circulating NSE was treatment responsive to the first chemotherapy cycle in patients with lung cancer, while inflammatory markers and vitamin D status were not significantly modified.

## 1. Introduction

Despite the generalized and sustained efforts made by the health authorities in recent years to help people acknowledge and avoid the most critical risk factors of lung cancer, this condition remained accountable for almost 2 million deaths in 2020 alone, according to the World Health Organization (WHO) [1]. Lung cancer is largely responsible for a high number of cancer-related deaths, and is also the second most prevalent type of neoplasm after skin cancer [2,3]. Considering the tiny fraction of patients diagnosed in their initial, localized phase, it is clear that both early diagnosis and treatment optimization should be considered when searching for solutions to combat cancer. To support early diagnostic capabilities, scientists developed novel treatments, trustful diagnostic and prophylactic tools [4]. Biomarkers obtained from blood, airway epithelial cells, or even from breath can be used nowadays to detect pulmonary cancer at early stages [5].

Genetic alterations were most commonly observed in lung adenocarcinomas. These findings can be useful for predicting the progression of carcinogenesis and even establishing personalized therapeutic targets. Furthermore, more effective classical remedies, chosen based on the specific tumoral drug- and/or radio-sensitivity, may be suggested [6]. Among these genetic alterations, which may contribute to lung cancer development, researchers have recently considered the gene encoding for the vitamin D receptor (VDR) [7,8]. Allowing the body to respond to the active form of vitamin D, its structural and functional modifications may indirectly lead to pulmonary pro-tumoral activity, since vitamin D is well known for its properties as an anticancer agent [9]. Several in vitro and in vivo experiments confirmed the relationship between this receptor and the development of lung cancer [10]. Particularly, recent studies suggested that a higher expression of VDR may be associated with better survival rates in lung cancer patients, while in some samples taken from lung tumoral sites, a lower expression of VDR was observed [11,12]. It is worth mentioning that elusive or inconclusive data were reported so far about the relationship between VDR and small cell lung cancer (SCLC), all the studies consulted being solely focused on the connection between VDR and non-small-cell lung carcinoma (NSCLC) [13].

As markers of macrophage activation, chitotriosidase (CHT) and neopterin are important players in the activation of immunological and inflammatory responses [14,15,16]. Circulating CHT is an enzyme mainly synthesized by neutrophils and activated macrophages [17]. Its immunomodulatory effects mainly involve the polarization of macrophages and the activation of cellular immunity [18]. Owing to its ability to hydrolyze chitin, chitotriose and chitobiose, CHT is largely known for its important role in human defense against various chitin-rich organisms [19,20]. Recognized for over two decades as a crucial biomarker in Gaucher’s disease, this enzyme was also linked to various other inflammatory disorders, such as atherosclerosis, diabetes mellitus and its complications, non-alcoholic fatty liver disease, several infections, stroke, chronic obstructive pulmonary disease, asthma, or overall lung injuries [21]. CHT, also called Chitinase 1, is encoded by the CHIT1 gene, and its increased enzymatic activity was previously associated with lung cancer [17,22]. However, most probably because of its implication in inflammatory reactions associated with lung conditions, this biomarker could not be safely used as a single discriminator between pulmonary neoplasm and benign lung inflammation [23,24]. In addition, there are several extensive reports in which chitinases and chitinase-like-proteins are considered obvious and important markers for different types of cancer and even targets for anti-angiogenic targeted cancer therapy [25,26].

As a catabolic compound of guanosine triphosphate (GTP), neopterin is largely acknowledged as a biomarker of cell-mediated immune-response activation [27]. An important indicator of viral infection, it has also been intensely studied as a prognostic biomarker in different types of cancer, such as pancreatic, breast, colorectal, and even lung cancer [28,29]. The significance of neopterin in lung cancer management was first observed more than a couple of decades ago [30]. Ever since, several other extensive studies have investigated the usefulness of this molecule as a tool for both diagnosis and prognosis, all of them confirming the initial theory, in which neopterin can be regarded as a trustful cancer biomarker [31]. There are more recent and intriguing studies, which show that while neopterin may be a circulatory biomarker of inflammation, it may also be regarded as an indicator of the mental state and fatigue in pulmonary cancer patients [32,33]. Thus, higher neopterin levels, associated with an augmented tryptophan breakdown biosignature, are strongly related to increased fatigue, considering their position as biomarkers of immune system activation [30].

Enolase 2 or neuron specific enolase (NSE) is a glycolytic enzyme that catalyzes the conversion of 2-phosphoglycerate to phosphoenolpyruvate [34]. It is found under several dimeric isoforms, containing three immunologically distinct subunits: α, β and γ. The mammalian α subunit of enolase is found in various tissues, while the β subunit is found mainly in the heart and skeletal muscle [35,36]. The αγ and γγ isoforms, which constitute neuron-specific enolase (NSE) or γ-enolase, are detected in high amounts in neurons and neuroendocrine cells, as well as in tumors originating from these cells [35,37]. NSE is currently the most reliable tumor marker in the prognosis of lung cancer evolution, and is recognized as a helpful tool for the differential diagnosis of SCLC and NSCLC [38,39].

Squamous cell carcinoma antigen (SCCA) is a cytoplasmic glycoprotein found in normal squamous epithelia [40]. In conjunction with clinical evaluation, SCCA assessment may serve as a non-specific tumor marker for the detection and monitoring of diverse squamous cell carcinomas, including those originating in the head and neck, esophagus, cervix, and lung [41,42].

Our pilot study aimed to investigate a possible association between the inflammatory status (reflected by circulating levels of CHT and neopterin) and vitamin D metabolism in lung cancer subjects. The usefulness of these biomarkers was assessed via comparison with the specific well-established tumoral markers, NSE and SCCA. Therefore, we conducted the first study to assess the modifications of circulating CHT, neopterin, NSE, SCCA, VDR, and 25-hydroxy vitamin D_3_ (25OHD_3_) before and after one cycle of chemotherapy. 

## 2. Materials and Methods

The study was conducted at the Amethyst Radiotherapy Center Cluj-Napoca and the Oncology Institute “Prof. Dr. Ion Chiricuță” Cluj-Napoca, Romania. All subjects included signed informed consent and the protocols were approved by the Ethics Committee of the University of Medicine and Pharmacy “Iuliu Hațieganu” Cluj-Napoca.

### 2.1. Inclusion Criteria

This observational study included twenty patients with lung cancer, consecutively selected from the medical files of the above-mentioned clinics in Cluj-Napoca. 

The diagnosis of lung cancer and its complications was established according to the Tumor, Node, Metastasis (TNM) staging system, thoracic computer tomography (CT) imaging, lung biopsy puncture, and pathological examination. The inclusion criteria were defined by the diagnosis of lung cancer, regardless of disease staging, before the first cycle of chemotherapy. After chemotherapy, patients were evaluated according to RECIST criteria 1.1, which is the gold standard for assessment of treatment response in solid tumors [43].

### 2.2. Exclusion Criteria

Age under eighteen and administration of vitamin D supplements, calcium, calcium antagonists, or corticosteroid drugs over the past six months were the main exclusion criteria. We also excluded immunocompromised patients.

### 2.3. Sample Preparation and Determination

Blood samples obtained from freshly drawn blood were centrifuged and stored at −80 °C. The second blood sample was obtained after seven days or three weeks from the first cycle of chemotherapy. An enzyme-linked immunosorbent assay (ELISA) for the quantification of serum levels of neopterin (FineTest, Wuhan, Hubei, China), and serum levels of NSE, SCCA, VDR and 25OHD_3_ (MyBioSource, San Diego, CA, USA) were performed according to the instructions of the manufacturers. Optical density was determined using a microplate reader (Stat Fax 2100 Awerness Technology, Palm City, FL, USA).

For all five parameters (neopterin, NSE, SCCA, 25OHD_3_, VDR), the intra-assay precision was ≤8% and the inter-assay precision was ≤10%.

The enzymatic activity of CHT was measured by a fluorometric method using an artificial substrate, according to the previously described method [44].

### 2.4. Statistical Analysis

Statistical analysis was performed in three different ways: for all patients (N = 20), for those who followed a seven-day treatment scheme, and for those who followed a three-week treatment scheme, as patients were stratified by the duration of the treatment. 

Continuous variables were presented as median and quartiles (Q1–Q3) or as means and standard deviations, and categorical variables as frequency and percentages. We performed descriptive and inferential statistics analysis to summarize the characteristics of the study population. The normality of distribution of quantitative variables was tested using the Shapiro–Wilk test. Its results showed a non-Gaussian distribution; therefore, we continued to use non-parametric tests. To compare the pre- and post-treatment values of specific laboratory markers, we employed the Wilcoxon signed-rank test.

To compare post-treatment values between the type of cancer and duration of the treatment, we employed Mann–Whitney U test. In order to evaluate the magnitude of the difference, we performed the effect size calculation for non-parametric distributions. The effect size (r) was calculated by dividing the Z score with the square root of subject number (Z/sqrt(N)). The common interpretation values for r in published literature are: 0.10– < 0.3 (small effect), 0.30– < 0.5 (moderate effect) and >= 0.5 (large effect).

Data analysis was performed using IBM SPSS v. 25.0 (Statistical Package for the Social Sciences, Chicago, IL, USA). The *p* value of <0.05 was considered to indicate a statistically significant difference. For a better understanding of our approach, we chose to present the median and quartiles and the mean and standard deviation.

## 3. Results

### 3.1. General Characteristics of the Study Group

The twenty patients included in our study had a median age of 64.5 [59.75–73.5] years, and most of them were men (80%). Male patients in this study were older than the female patients, but no significant statistical difference was observed (65.5 vs. 59 years, *p* = 0.178). Four patients (20%) were non-smokers, five (25%) were former smokers, and eleven (55%) were current cigarette smokers. There were no statistically significant differences in gender distribution according to the smoking status (*p* = 0.958).

Regarding the stage of the disease before treatment, most subjects presented stage III non-small-cell lung cancer, which is divided into three different stages: IIIA (1 patient, 5%), IIIB (9 patients, 45%) and IIIC (1 patient, 5%). 

Using RECIST 1.1 criteria, we have likened the evolution of the remaining fifteen lung cancer patients after chemotherapeutic treatment (five had to be excluded due to a lack of available data). Therefore, one patient showed complete remission, one had complete remission with persistent imaging abnormalities, four had a partial response, eight had minimal disease progression, and only one had a significant progression of the disease.

With respect to the histopathological type of cancer, the majority of the patients had adenocarcinoma (ten patients, 50%), followed by squamous cell carcinoma (six patients, 30%), and small-cell lung cancer (four patients, 20%).

### 3.2. Biochemical Parameters

We assessed the general status of the enrolled patients before and after the chemotherapeutic treatment.

Baseline and post-treatment values of the specific biological markers are summarized in Table 1. Pre- and post-treatment values of CHT, VDR and 25OHD_3_ did not show statistically significant variations. Post-treatment values of NSE were significantly lower compared to pre-treatment values (14.37 vs. 17.10 ng/mL, *p* = 0.031). A similar trend in neopterin levels could be noticed, but the difference was only marginally significant (1.44 vs. 1.17 ng/mL, *p* = 0.069). On the contrary, the variations of circulating SCCA, CHT, neopterin, VDR, and 25OHD_3_, before and after treatment, did not reach statistical significance. 

Even there were not statistically significant differences, we observed a medium effect size between pre- and post-treatment values for Neopterin (r = 0.326) and NSE (r = 0.459), while for the rest there was a small effect size.

Post-treatment values of the specific laboratory markers were further stratified by the duration of treatment, and the results are presented in Table 2. In all six analyzed markers, we did not find any significant difference between patients who followed a seven-day treatment scheme compared to those who followed the three-week treatment scheme.

Finally, when we compared post-treatment values of the specific laboratory markers by the type of cancer, we did not find any statistically significant differences. These results are summarized in Table 3a,b.

Table 3The post-treatment values of specific laboratory stratified by cancer type.

**Table:** (**a**)

	Adenocarcinoma (N = 10)	Small Cell Lung Cancer and Squamous Cell Carcinoma (N = 10)	*p* Value	Effect Size (r)
CHT (nmol/mL/h) post-treatment	285.00 (213.75, 347.50)	212.50 (192.50, 340.00)	0.448	0.170 (s)
25OHD_3_ (nmol/L) post-treatment	55.03 (50.16, 66.96)	56.80 (53.66, 63.87)	0.940	0.016 (s)
Neopterin (ng/mL) post-treatment	1.18 (1.05, 1.22)	1.07 (0.86, 1.14)	0.344	0.211 (s)
VDR (ng/mL) post-treatment	0.27 (0.08, 0.52)	0.32 (0.25, 0.48)	0.761	0.060 (s)
NSE (ng/mL) post-treatment	11.74 (8.41, 13.79)	11.95 (9.93, 15.63)	1.000	0 (s)
SCCA (ng/mL) post-treatment	0.01 (0.00, 0.02)	0.06 (0.00, 0.10)	0.095	0.373 (m)

Abbreviations: Legend: CHT—chitotriosidase; NSE—neuron specific enolase; SCCA—squamous cell carcinoma antigen; VDR—vitamin D receptor; 25OHD_3_—25-hydroxy vitamin D_3_; Data are presented as median and quartiles (Q1-Q3), Mann–Whitney U test. r—effect size; s—small; m—moderate; l—large.

**Table:** (**b**)

	Adenocarcinoma (N = 10)	Small Cell Lung Cancer (N = 4)	Squamous cell carcinoma (N = 6)	*p* Value	Effect Size (r)
	Median (Q_1_, Q_3_)	Median (Q_1_, Q_3_)	Median (Q_1_, Q_3_)		
CHT (nmol/mL/h)	285.0 (213.75, 347.50)	180.00 (120.0, 206.25)	330.0 (227.50, 350.0)	0.448	0.170 (s)
Neopterin (ng/mL)	1.18 (1.05, 1.22)	0.96 (0.81, 1.09)	1.10 (0.95, 1.18)	0.344	0.211 (s)
NSE (ng/mL)	55.55 (13.54, 95.57)	19.57 (10.46, 60.80)	14.27 (10.75, 44.54)	1.000	0 (s)
SCCA (ng/mL)	0.03 (0.0, 0.10)	0.18 (0.08, 0.58)	0.08 (0.02, 0.10)	0.095	0.373 (m)
VDR (ng/mL)	0.27 (0.08, 0.52)	0.25 (0.25, 0.36)	0.40 (0.19, 0.48)	0.761	0.060 (s)
25OHD_3_ (nmol/L)	55.03 (50.16, 66.96)	56.80 (49.33, 62.41)	56.71 (53.66, 63.87)	0.940	0.016 (s)

Abbreviations: CHT—chitotriosidase; NSE—neuron specific enolase; SCCA—squamous cell carcinoma antigen; VDR—vitamin D receptor; 25OHD_3_—25-hydroxy vitamin D_3_; data are presented as median and quartiles (Q1–Q3), Mann–Whitney U test. r—effect size; s—small; m—moderate, l—large.

## 4. Discussion

The serum concentration of tumor markers (TMs) produced by tumor cells is useful for early diagnosis of cancer, the assessment of tumor volume, disease extent and response to treatment. TMs may also have a prognostic value, as they can represent indicators of recurrences.

TMs have been intensively studied in lung cancer as a non-invasive tool for differentiating between SCLC and NSCLC prior to biopsy or surgery, and still represent a priority in this research field [41].

While NSE is widely accepted as a biomarker in SCLC, its usefulness in NSCLC is not clearly defined yet. Circulating NSE levels illustrate statistically significant variations in SCLC according to the status of patient: elevated pre-treatment levels will initially increase after chemotherapy (tumor lysis syndrome), followed by a decrease in responder patients (chemotherapy sensitive tumors) [45]. In SCLC, a recent study by Li et al. [46] showed the prognostic value of NSE levels: elevated baseline and three-week levels were associated with worse prognosis in advanced SCLC patients receiving immunotherapy [46]. On the other hand, in NSCLC patients, a recent study by Yan et al. [47] found increased NSE serum levels in 30–69% of patients, but still little is known about the clinical evolution of NSE in this type of lung cancer [47].

Moreover, circulating NSE could also represent a useful marker in NSCLC and a significant and independent predictor of survival. According to the studied literature, patients with lower NSE pre-treatment levels have a significantly higher survival rate than those with elevated levels, and the prognostic value of NSE is independent of other prognostic variables [45].

In our study, only serum levels of NSE significantly increased after the first cycle of chemotherapy (Table 1). Of the twenty patients enrolled in this pilot study, thirteen had higher post-treatment values, but seven patients presented a small decrease in NSE levels. In the latter cases, we may postulate that the first cycle of chemotherapy had an initial favorable effect (small tumor reduction). Most of the patients had higher post-treatment NSE levels, suggesting that chemotherapy may cause an increase in circulating NSE through tumor lysis syndrome. If we corroborate the cancer type (Table 3a,b), the post-treatment NSE concentrations were highest in patients with SCLC, followed by NSCLC patients (adenocarcinoma), and the smallest increases were registered in squamous carcinoma cases. A possible explanation could be that adenocarcinoma patients responded better to chemotherapy than SCLC patients. This observation is difficult to interpret given the limited data available in the literature on post-treatment NSE levels in patients with NSLC and its predictive power. A study conducted by Dal et.al [48] on CEA, CYFRA 21-1 and NSE levels in patients with advanced NSLC (54 out of 70 patients presented with adenocarcinoma) found no significant modifications in NSE serum levels after chemotherapy treatment [48]. In their study conducted on serum mRNA levels of NSE in advanced NSLCL patients, Wang et al. [49] found a statistically significant correlation between successful gefitinib-based chemotherapy and low NSE mRNA levels. Xu et al. [50] concluded that increased NSE levels in adenocarcinoma patients are correlated with oncogene mutation, which in turn can lead to drug resistance, tumor transformation, and ultimately poor prognosis in these patients [50].

Our study provides supplementary data on NSE usefulness as a serum TM in lung cancer treatment response. As a preliminary study, we found significant results in patients with NSCLC, sustaining the value of NSE as a follow-up marker and independent prognostic factor in lung cancer treatment.

Regarding neopterin, its increased concentrations in serum or in the tumor microenvironment correlate with phenotypic and functional changes of lymphocytes, indicating immune dysfunction [51]. In the evolution of lung cancer, the inflammation and the suppression of cellular immunity play important roles; therefore, neopterin may be viewed as a marker of immune activation and inflammation and could be used as an indicator of lung cancer progression and treatment responsiveness [52].

Data from studied literature regarding the correlation of serum neopterin with the lung cancer type are contradictory. In patients with lung cancer, the increase in serum neopterin levels was found to be dependent on the tumor type and disease stage: mean values were higher in patients with NSCLC (adenocarcinoma) stage IV compared to stages I to III [53,54]. Other studies sustained that serum neopterin levels were significantly higher in patients with SCLC than those with adenocarcinoma [55].

In search of a useful TM in lung cancer, data from older studies could not document the value of neopterin as an adjuvant parameter in assessing radiotherapy and chemotherapy effects in lung cancer, or other types of cancer [56]. Additional studies claimed that increased serum levels of neopterin reflects the efficacy of anticancer immunotherapy [57,58] or chemotherapy [58], the assessment of anticancer treatment side effects, the prediction of subsequent complications, and poor prognosis [30].

In our study, the median values of neopterin presented slight differences according to the cancer type (Table 3a,b); they were more elevated in NSCLC (adenocarcinoma) and SCLC but decreased in squamous carcinoma. Additionally, we observed a statistically significant decrease in neopterin levels after chemotherapy (Tabel 1). Neopterin can serve as an immunologically estimation of malignant outgrowth [59], and an association between higher baseline serum neopterin ratio and toxicity of therapy has been previously observed [60]. Contrary to our results, Melichar et al. [61] reported increased neopterin levels after administration of neoadjuvant chemotherapy in patients with breast carcinoma. They attributed this occurrence to systemic inflammation (release of pro-inflammatory cytokines) and immune activation, further linking the rise in neopterin to observed chemotherapy side-effects (flu-like symptoms and fatigue). Similarly, Holečková et al. [62] found that in patients with head and neck carcinoma receiving radiotherapy, neopterin serum levels increased during and post-treatment and were closely associated with manifestation of treatment toxicity [62].

Regarding our results, we can hypothesize that the decrease in neopterin serum levels in squamous carcinoma patients could be due to the administration of a better tolerated chemotherapeutic agent which brought on an initial better response (tumor reduction) in these patients.

According to recent studies [63], vitamin D has a protective and antiproliferative effect on lung cancer. In fact, the whole vitamin D axis key elements, including vitamin D binding protein, could be altered and thus influence the prognosis of lung cancer [64]. Several studies have shown that patients with low levels of vitamin D may be more prone to developing lung cancer [65]; moreover, low levels of vitamin D and the variation of VDR serum levels were also found in lung cancer, as well as in other cancer types [66]. It is possible that VDR polymorphisms represent a genetic susceptibility factor for lung cancer, but further studies should be conducted to validate this association [65].

Furthermore, vitamin D_3_ levels are modulated by VDR genetic variation and are inversely correlated with lung cancer status [67]. The association between vitamin D/VDR status and lung cancer depends on tumor type and staging. Increased VDR expression in lung adenocarcinoma is associated with improved survival [68]. Other studies and meta-analyses sustained contradictory data, indicating that vitamin D_3_ does not predict cancer survival rate and that VDR expression is variable in tumors [66,69]. In patients with advanced NSCLC, a study by Heist et al. [70] did not find a significant effect of vitamin D_3_ level on overall survival, but various VDR polymorphisms were associated with worse survival rates [70]. Our study was consistent with Heist et al. [70], finding circulating vitamin D_3_ and VDR levels with very slight decreases after the first cycle of chemotherapy that were not statistically significant (Table 1). Compared with vitamin D_2_, vitamin D_3_ is more strongly associated with lung cancer, but in our study, the detection method used is less sensitive, as many immunoassays suffer from cross-reactivity between vitamin D_2_ and D_3_ forms, therefore resulting in confounded measurements [67]. However, since the number of patients included in the present study was relatively small, with patients presenting normal baseline levels of vitamin D_3_ (upper limit) and as the time between sample collection pre- and post-treatment was relatively short (three weeks) we cannot rule out the possibility of level modifications in time. Further studies on a larger sample size, taken continuously over the period of the treatment, would offer much-needed information on the evolution of vitamin D_3_ and VDR levels and their post-treatment prediction power on lung cancer patient outcomes.

CHIT1, mainly produced by activating macrophages, both in normal and inflammatory conditions [71], could represent a promising biomarker correlated with disease activity, severity, and extent in patients with several pulmonary infections and diseases with an inflammatory component [72], including lung cancer [73].

Our study showed no statistical variation in circulating CHIT1 between pre- and post-treatment values (Table 1). The analysis of CHIT1 mean values according to cancer type (Table 3a,b) indicated various concentration differences, with highest levels in squamous carcinoma, lower levels in the case of NSCLC (adenocarcinoma and the smallest recorded in SCLC. However, the differences did not reach statistical significance. The appearance of an inflammatory microenvironment in tumors is driven by different events. In the tumor microenvironment, the inflammation could be amplified by the chemotherapy effect. In our study, NSE and CHT evolved in the same direction, increasing after chemotherapy, a fact that can be interpreted as a chemotherapeutic induction of acute inflammatory reactions, activating anti-tumor immune responses [72].

With respect to SCCA, its serum levels are higher in lung cancer patients. As such, SCCA serum level is an important marker in the diagnosis and therapeutic monitoring of lung squamous carcinoma [73,74].

According to cancer type (Table 3a,b), SCCA had the highest values in SCLC, followed by squamous carcinoma, and were lowest in adenocarcinoma, without reaching statistical significance. SCCA levels vary in lung cancer, depending on the morphological type, but literature data are inconsistent. Similar to our study, SCCA values are higher in SCLC patients than in squamous cell and adenocarcinoma lung cancer patients [75]. Increased SCCA serum levels have a prognostic value, especially in squamous cell carcinomas, but we had fewer cases of squamous carcinoma patients. Moreover, increased serum SCCA levels have also been observed in other inflammatory and malignant diseases, such as adenocarcinoma, hepatocarcinoma, or kidney disease, indicating that squamous cell carcinoma is not the only originating source of serum SCCA [76]. In small-cell lung cancer patients, higher SCCA level, clinical stage and the number of chemotherapy cycles (more than four) are independent prognostic factors correlated with a good progression-free survival [49]. This is similar to the post-treatment SCCA levels that increased mostly in SCLC patients, indicating a good response from these patients, even after one cycle of chemotherapy.

Certain limitations of our study should be considered. First, the sample size was relatively small, and the study design was observational. Another potential limitation was that serum CHT, neopterin, NSE, SCCA, VDR, and 25OHD_3_ levels were only assessed after the first cycle of chemotherapy. Different stages of the disease may also influence the serum concentration of these parameters after the first dose of chemotherapy. Further studies are required for the evaluation of these parameters after several cycles of chemotherapy.

However, our data should be considered preliminary results that could open new research opportunities. To our knowledge, the present study is the first to evaluate the serum levels of CHT, neopterin, NSE, SCCA, VDR, and 25OHD_3_ in lung cancer patients after the initial cycle of chemotherapy.

## 5. Conclusions

The study did not concentrate on assessing the variations of tumor markers, inflammation status, and vitamin D metabolism in lung cancer subjects compared with the general population, but to highlight pre- and post-treatment variation of these biomarkers and to issue possible mechanisms that lie behind their modifications.

In patients with lung cancer, serum levels of NSE were significantly decreased after the first chemotherapy cycle compared with pre-treatment values. In addition, the post-treatment values of neopterin were lower as well, but with marginal statistical significance. Conversely, circulating levels of CHT, SCCA, vitamin D, and VDR were not significantly modified in response to the first chemotherapy cycle. Therefore, in patients with lung cancer, the combined detection of several biomarkers (inflammatory and cell surface membrane antigens) can improve the staging and the evaluation for treatment response.

## Figures and Tables

**Table 1:** Baseline and post-treatment characteristics of the studied biomarkers.

	Pre-Treatment (N = 20)	Post-Treatment (N = 20)	*p* Value	Effect Size (r)
CHT (nmol/mL/h)	240 (205, 305)	242.50 (197.50, 350)	0.337	0.129 (s)
Neopterin (ng/mL)	1.20 (0.94, 1.79)	1.10 (0.91, 1.20)	0.069	0.326 (m)
NSE (ng/mL)	15.84 (12.99, 19.72)	21.89 (11.79, 84.12)	0.031	0.459 (m)
SCCA (ng/mL)	0.02 (0.0, 0.09)	0.08 (0.0, 0.10)	0.136	0.101 (s)
VDR (ng/mL)	0.29 (0.11, 0.43)	0.27 (0.12, 0.51)	0.284	0.147 (s)
25OHD_3_ (nmol/L)	56.62 (44.63, 62.72)	55.74 (52.01, 66.12)	0.894	0.025 (s)

Abbreviations: CHT—chitotriosidase; NSE—neuron specific enolase; SCCA—squamous cell carcinoma antigen; VDR—vitamin D receptor; 25OHD_3_—25-hydroxy vitamin D_3_. Data are presented as median and quartiles (Q1–Q3), Wilcoxon signed-rank test. r—effect size; s—small; m—moderate; l—large.

**Table 2:** The post-treatment values of a specific laboratory stratified by treatment duration.

	1–7 Days (N = 6)	14–21 Days (N = 14)	*p* Value	Effect Size (r)
CHT (nmol/mL/h) post-treatment	267.50 (176.25, 340.0)	242.50 (200, 347.50)	0.772	0.064 (s)
Neopterin (ng/mL) post-treatment	0.99 (0.86, 1.11)	1.15 (1.05, 1.20)	0.409	0.185 (s)
NSE (ng/mL) post-treatment	20.91 (10.82, 47.86)	26.12 (12.23, 95.57)	0.409	0.018 (s)
SCCA (ng/mL) post-treatment	0.18 (0.02, 0.37)	0.06 (0.0, 0.10)	0.269	0.303 (m)
VDR (ng/mL) post-treatment	0.33 (0.25, 0.61)	0.27 (0.09, 0.49)	0.709	0.083 (s)
25OHD_3_ (nmol/L) post-treatment	55.74 (53.66, 64.04)	56.45 (50.16, 64.58)	1.000	0 (s)

Abbreviations: CHT—chitotriosidase; NSE—neuron-specific enolase; SCCA—squamous cell carcinoma antigen; VDR—vitamin D receptor; 25OHD_3_—25-hydroxy vitamin D_3_; data are presented as median and quartiles (Q1-Q3), Mann–Whitney U test. r—effect size; s—small; m—moderate; l—large.

## Data Availability

Not applicable.
